# HCV Pit Stop at the Lipid Droplet: Refuel Lipids and Put on a Lipoprotein Coat before Exit

**DOI:** 10.3390/cells8030233

**Published:** 2019-03-12

**Authors:** Gabrielle Vieyres, Thomas Pietschmann

**Affiliations:** 1 Institute of Experimental Virology, TWINCORE, Centre for Experimental and Clinical Infection Research, a Joint Venture between the Medical School Hannover (MHH) and the Helmholtz Centre for Infection Research (HZI), 30625 Hannover, Germany; 2 German Centre for Infection Research (DZIF), Partner Site Hannover-Braunschweig, 38124 Braunschweig, Germany

**Keywords:** hepatitis C virus, virus assembly and release, lipoprotein, VLDL, lipid droplet, apolipoprotein, lipolysis, DGAT, membrane contact sites, molecular mimicry

## Abstract

The replication cycle of the liver-tropic hepatitis C virus (HCV) is tightly connected to the host lipid metabolism, during the virus entry, replication, assembly and egress stages, but also while the virus circulates in the bloodstream. This interplay coins viral particle properties, governs viral cell tropism, and facilitates immune evasion. This review summarizes our knowledge of these interactions focusing on the late steps of the virus replication cycle. It builds on our understanding of the cell biology of lipid droplets and the biosynthesis of liver lipoproteins and attempts to explain how HCV hijacks these organelles and pathways to assemble its lipo-viro-particles. In particular, this review describes (i) the mechanisms of viral protein translocation to and from the lipid droplet surface and the orchestration of an interface between replication and assembly complexes, (ii) the importance of the triglyceride mobilization from the lipid droplets for HCV assembly, (iii) the interplay between HCV and the lipoprotein synthesis pathway including the role played by apolipoproteins in virion assembly, and finally (iv) the consequences of these complex virus–host interactions on the virion composition and its biophysical properties. The wealth of data accumulated in the past years on the role of the lipid metabolism in HCV assembly and its imprint on the virion properties will guide vaccine design efforts and reinforce our understanding of the hepatic lipid metabolism in health and disease.

## 1. Introduction

Mix oil and water, let it stand, and the mixture segregates into two phases; a light oil fraction overlaying the heavier water. Upon shaking, an unstable emulsion is created with droplets of lipids dispersed in the aqueous solution, whereby the contact surface between the two phases increases. Adding a drop of detergent stabilizes the emulsion. Within eukaryotic cells, “oily” neutral lipids serve as the main energy source. However, due to their hydrophobic nature, within the aqueous environment of cells, neutral lipids are embedded in a monolayer of amphipathic lipids, mostly phospholipids, which serve as a detergent and stabilize an emulsion of lipid droplets within the cytoplasm of cells. Proteins are targeted to these organelles in a dynamic manner, mediating their transport, anabolism, and catabolism to serve the needs of cells and their organelles [1]. Lipid droplets are ubiquitous in all cells and tissues, however, the adipose tissue, the skeletal muscles and the liver are the top three players in fatty acid metabolism [2]. The adipose tissue has the main neutral lipid storage capacity. It stores fatty acids or dispenses them as albumin-bound non-esterified fatty acids in the circulation for the other tissues of the organisms in response to the nutritional state. Heart and skeletal muscles are the major oxidative tissues and typically feed fatty acids into β-oxidation within the mitochondria for energy production. As for the liver, with all its metabolic functions, it comprises a specialized machinery for the production and secretion of lipoproteins, which serve to transport various lipid species via the bloodstream to distant sites and tissues in the organism: triglycerides from the lipid droplets are hydrolyzed into fatty acids and re-esterified within the endoplasmic reticulum (ER) in triacylglycerides (TAGs) that are packaged into very-low-density lipoproteins (VLDLs). Lipid droplets (LDs) are also a reservoir for membrane lipid precursors and a source of signaling lipids [3]. Multiple human pathologies with abnormally low fat storage (e.g., lipodystrophies and cachexia) or aberrant fat accumulation (e.g., neutral lipid storage diseases, nonalcoholic fatty liver disease, atherosclerosis, or metabolic syndrome) can be traced back or associated with genetic deficiencies in the lipid droplet metabolic pathway [4]. The variety of functions of lipid droplets also makes them an attractive target for pathogens to sustain their replication. Thus, several viruses, including HCV, dengue virus, rotaviruses, but also a number of bacteria and parasites exploit this organelle to complete different steps of their multiplication [5].

HCV is a hepatotropic human pathogen. Its compact genome encodes 10 proteins [6]. Half of them, the non-structural (NS) proteins NS3, 4A, 4B, 5A, and 5B form the replication complexes around the NS5B RNA-dependent RNA-polymerase that copies the viral genome. Like other positive-strand RNA viruses, HCV replicates its genome on modified intracellular membrane, in this case on a so-called “membranous web” that originates from the endoplasmic reticulum [6]. A narrow cooperation between all viral proteins, including the structural proteins core (capsid), E1 and E2 (envelope glycoproteins), the p7 viroporin and the non-structural proteins NS2 and NS3-5B is necessary for virion morphogenesis [6]. HCV has also established an intricate network of molecular interactions with its host cell that sustains all steps of its replication cycle. In particular, it adapted and attuned its replication strategy to the liver landscape. Thus, HCV uses a combination of entry factors including lipid and lipoprotein receptors that is quite unique to the hepatocyte (e.g., Claudin-1, Scavenger Receptor class B type I (SR-BI), low density lipoprotein receptor (LDL-R), CD81 and Occludin) [7]. HCV RNA also contains 2 binding sites for the liver-specific miR-122, which is an essential cofactor for its translation and replication [8]. Finally, by hijacking the lipid droplets, HCV gets access to the VLDL biogenesis and uses part of this pathway for its morphogenesis. This review summarizes these peculiar host-virus interactions and their impact on the virion biophysical properties.

## 2. A Chip off the Old Block: HCV Particle Properties Reflect Interactions with the Lipoprotein Production Pathway

### 2.1. HCV Particles Resemble Host Serum Lipoproteins

One striking property of HCV virions is their low buyoant density, with infectious particles peaking at 1.03–1.10 g/mL [9]. This feature sets HCV apart from most other enveloped viruses and is rather reminiscent of serum lipoproteins, as summarized before [9]. Interestingly, HCV virion density is influenced by dietary triglycerides in vivo and depends on the host cell system used for virus production, thus highlighting that host cellular processes coin particle properties [10,11]. Typically, HCV grown in cultured cell lines (HCVcc) can infect animals such as chimpanzees and human liver chimeric mice but the virus recovered from these animals has a lower buoyant density and an increased infectivity as compared to the cell culture-derived inoculum [12]. *Vice versa*, HCV grown in primary human hepatocytes displays a lower buoyant density and higher specific infectivity than HCVcc but this characteristic is lost once this virus is cultured in cell lines [10]. These observations suggest that host factors whose abundance or functions differ between hepatoma cell lines and authentic liver cells are responsible for the atypical virion density profile.

**Figure 1:**
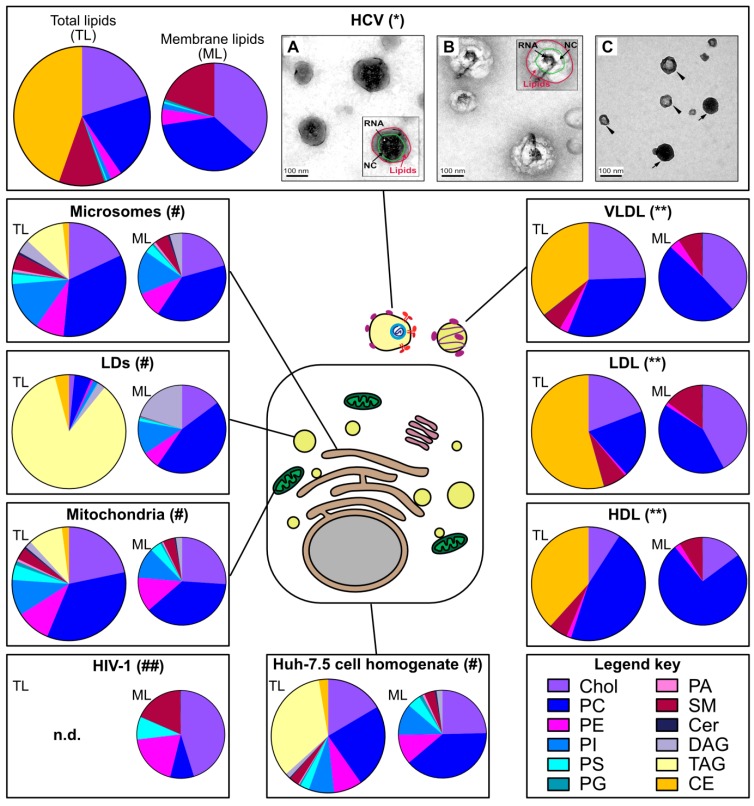
Hepatitis C virus (HCV) atypical lipid profile and morphology mimic serum lipoproteins. Within the top panel, (**A**) and (**B**) are electron micrographs of HCV grown in cultured cell lines (HCVcc) (**A**) and human serum-derived HCV (**B**) captured on electron microscopy grids with anti-E2 antibodies. Picture (**C**) was obtained by immunocapturing particles from the culture supernatant of HCVcc-producing cells with anti-ApoE antibodies. Arrows point at putative HCV lipo-viro-particles while arrowheads indicate presumed lipoprotein particles. Pictures (**A**), (**B**) and (**C**) were kindly provided by J.-C. Meunier, Université de Tours, France (unpublished material related to Piver E. et al. [17]).The different panels of the figure represent the lipid composition of different Huh-7.5 subcellular fractions (#) [21], as well as affinity purified Flag-tagged HCVcc (*) [15], velocity gradient purified HIV-1 virions (##) [34], and serum lipoproteins (**) [35]. In each panel, the left bigger pie chart represents all detected lipid species (total lipids = TL), whereas the right smaller chart focuses on the subset of membrane lipids (membrane lipids = ML). The comparison of the lipidomes of HCV and the microsomes, the latter comprising the ER which is the putative budding site of HCV, indicates a dramatic enrichment of neutral lipids in HCV particles (depicted in yellow), even though the TAGs could not be quantified for HCV in the source publication [15]. The individual membrane lipids also deviate from the host cell membranes. In contrast, the HCV lipidome resembles rather the ones of LDL and VLDL (right panels). Note that also different experimental conditions used in the four cited original studies might in part account for the detection of different lipid species. For example, triacylglycerides (TAGs) were not quantified in the HCV [15] and lipoprotein [35] lipidomes, and the HIV-1 proteome focused on membrane lipids [34] (hence the omission of the left pie chart). Therefore, we refer the interested reader to the original articles in order to find the details of the quantified lipids. Chol, cholesterol; PC, phosphatidylcholine; PE, phosphatidylethanolamine; PI, phosphatidylinositol; PS, phosphatidylserine; PG, phosphatidylglycerol; PA, phosphatidic acid; SM, sphingomyelin; Cer, ceramides; DAG, diglycerides; TAG, triglycerides; CE, cholesterol ester. The lipid species are displayed clockwise in the pie chart according to their order in the legend key.

While HCV’s peculiar density was described very early, the virion morphology and composition has remained a puzzle for decades. Electron microscopy is still a key technique in virus discovery and diagnostic [13], but in the case of HCV, 30 years after the virus discovery, only a few rather recent studies could convincingly image the virion in cell culture supernatants and in patient serum (e.g., [14,15,16,17]) (Figure 1). These studies mostly used potent antibodies directed against natural epitopes or engineered tags in HCV glycoproteins or against apolipoproteins to directly capture virions from high-titer virus preparations on affinity electron microscopy grids [16,17]. Globally, this laborious endeavor taught the following lessons: (i) HCV virion is heterogenous in size, in a range from 40 to 140 nm [16,17], roughly in line with filtration and sedimentation velocity gradient experiments [9,18], (ii) comparable to serum lipoproteins, this size is dramatically reduced after delipidation [14,17]; (iii) HCV has no apparent symmetry, neither for its envelope nor for its capsid [14,15,16,17], this is in contrast to the well-arranged flavivirus relatives [9]; (iv) HCV displays not only its envelope glycoproteins at its surface but also a range of apolipoproteins (see below) [14,15,16,17].

### 2.2. HCV Lipidome Exhibits an Excess of Neutral Lipids

The virion lipidome can give a useful hint on the site of virus assembly. For instance, the lipidomes of vesicular stomatitis virus and Semliki forest virus strictly reflect the composition of the cell plasma membrane from which they bud [19]. As another example, egress of human cytomegalovirus requires the SNARE machinery and the virion lipidome resembles synaptic vesicles [20]. Merz and colleagues reported the lipidome of affinity-purified HCVcc [15] (Figure 1). One striking observation was the high ratio of the apolar (in this case the cholesteryl esters, as the triglycerides were not monitored in this study) *versus* polar lipids (e.g., phospholipids). This low proportion of membrane lipids is incompatible with the structure of a canonical enveloped virion and suggests the incorporation of a neutral lipid core within or attached to the particle. Furthermore, the HCV virion lipidome does not only differ from the global lipid composition of the Huh-7.5 host cell, it is also discrepant with the ER membrane composition [21], the putative site of HCV assembly (see below, Section 4). Rather, the HCV lipid landscape is hardly distinguishable from that of low and very-low-density lipoproteins [15] (Figure 1).

### 2.3. Apolipoproteins Make an Important Part of the Virion Proteome

Incorporation of host cell proteins is common during virus morphogenesis [22]. In the case of HCV, in addition to the three viral structural proteins, a range of apolipoproteins are incorporated within the virion envelope and actually participate in virion entry and in protecting the virus against antibody-mediated neutralization [23]. These apolipoproteins include ApoB and the exchangeable apolipoproteins ApoA-I, ApoC-I, ApoC-II, ApoC-III and ApoE [23]. Several lines of evidence including virion immunopurification with anti-apolipoprotein antibodies [15,24,25], virion immunogold labelling [14,15,16,17], neutralization of HCV entry by anti-apolipoprotein antibodies [15,25,26] and also detection of apolipoproteins by mass spectrometry on immunopurified virions [15,16,27] firmly support the conclusion that apolipoproteins are part of HCV particles. In addition, several proteins involved in the host lipid metabolism were detected among the 46 virion-associated proteins identified in a proteomics approach [27]. Altogether, the biophysics and the biochemical composition of HCV virion suggest a peculiar virus assembly process tightly relying on the host cell lipoprotein machinery.

### 2.4. Several HCV Proteins Colocalize with Lipid Droplets

The direct association between HCV particles and lipoproteins suggests that the virus might follow the lipoprotein secretion pathway. Consistent with this notion, tetracysteine-tagged core protein traffics together with GFP-tagged ApoE in infected cells [28]. More strikingly, a number of HCV proteins accumulate at the surface of the lipid droplets, the intracellular source of lipids for the VLDL production. This observation, first reported for ectopically expressed core protein and at the time often regarded as an artefact [29], was later confirmed in the HCVcc system [30,31,32]. Not only core but also several non-structural proteins, such as NS3 and NS5A were detected in a ring pattern around the lipid droplets [30,31] (see Section 3.2.2). The rest of this review will summarize how HCV accesses the lipid droplet organelle and how we think this first step in virus assembly enables the virus to engage the lipoprotein production pathway, resulting in the production of a lipo-viro-particle [33] rather than a canonical enveloped virion.

## 3. From the ER, HCV Takes a Grip on the Lipid Droplet: Building an Interface between Replication and Assembly Complexes

### 3.1. Structural Basis for the Association of HCV Proteins with the Lipid Droplet Monolayer

#### 3.1.1. The Physiological Case: Several Ways to Bind a Lipid Droplet

**Figure 2:**
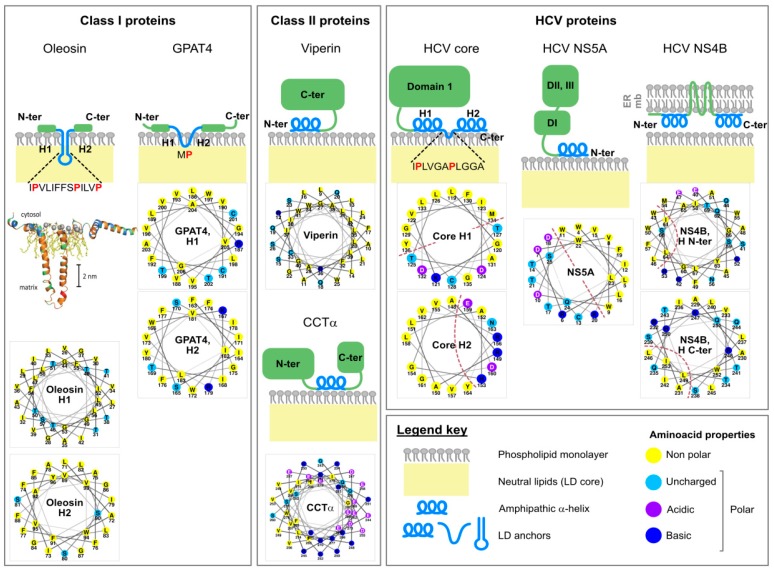
Different ways to bind lipid droplets. Presumed topology of representative host and viral lipid droplet-binding proteins: *P. patens* plant oleosin, drosophila GPAT4 [39], mouse viperin [49], human CCTα [57], HCV core (genotype 1a strain Glasgow) [45], NS5A (consensus sequence) [47], NS4B (genotype 1b strain O) [56]. Wheel representations of the predicted or confirmed α-helices were drawn with Netwheels (http://lbqp.unb.br/NetWheels/) [58]. Dashed brown lines where assigned by the authors (where relevant) and indicate the boundary between hydrophobic and hydrophilic portion of the helix. The secondary structure of oleosin represented on the lipid droplet surface was based on homology modeling and is reproduced from Huang et al. [38] with permission of the authors and of the American Society of Plant Biologists (permission obtained via Copyright Clearance Center).

The phospholipid monolayer delimitating the lipid droplet imposes constraints for protein targeting to this organelle [36]. Although some proteins bind lipid droplets indirectly via protein-protein interactions or a lipid anchor, most are targeted by structural elements present in their protein sequence. Depending on their origin, these proteins can be assigned into two categories, as summarized by Kory and colleagues [36] (Figure 2).

Class I proteins are translated at the ER and localize both to this compartment and to the LD surface. They contain a hydrophobic sequence, without any ER luminal domain. Mostly, the hydrophobic sequence is predicted to form a hairpin, with two α-helical domains. At the tip of the hairpin, 2 or 3 proline residues often form a “proline knot” that might break the helix and insert either between the two membrane leaflets of the ER or in the LD hydrophobic core. Plant oleosins are the best known examples of this category; proteinase K mapping experiments support the hairpin structure of their lipid droplet-binding domain [37], and homology modeling predicts its mostly helical fold [38]. However, other proteins such as GPAT4 (glycerol-3-phosphate acyltransferase 4) [39] and DGAT2 (acyl-CoA:diacylglycerol O-acyltransferase 2), which both participate in lipid droplet expansion [39], and also AUP1 (ancient ubiquitous protein 1) [40] might also use such an anchor. These proteins indeed display a motif of ~40 residues with two predicted hydrophobic helices separated by a short hydrophobic sequence [36]. In the case of oleosins, the exceptional length of the hydrophobic hairpin (~11 nm or ~70 residues) is incompatible with the thickness of a phospholipid monolayer and is believed to insert in the lipid droplet neutral lipid core [41]. This topology could favor the concentration of oleosins at the sites of lipid droplet neogenesis at the ER, where triglycerides accumulate between the two leaflets of the ER membrane, providing more space to accommodate the extensive oleosin hairpin. This local concentration of oleosins at the LD nucleation sites may, in turn, facilitate their translocation from the ER to the lipid droplet surface.

Class II proteins are translated in the cytosol and adsorb to the droplet surface via amphipathic helices or hydrophobic domains. A representative member of this second class is for instance, CCTα (CTP:phosphocholine cytidylyltransferase α), the rate-limiting enzyme of the Kennedy pathway of phosphatidylcholine synthesis. Moreover, several PAT (perilipin/ADRP/TIP47)-domain proteins including ADRP (PLIN2) and TIP47 (PLIN3) harbor a central 11-mer repeat-containing domain predicted to fold into amphipathic and hydrophobic helices upon membrane binding. The C-terminal four-helix bundle of ADRP and TIP47 furthermore participates in lipid droplet binding. Interestingly, 11-mer repeats and helix bundles also maintain apolipoproteins on the lipoprotein surface (see Section 4.4). It is not clear, however, how class II proteins distinguish the LD surfaces from other intracellular membranes.

#### 3.1.2. HCV Dispatches Some of Its Proteins onto the LD Surface

Upon translation, the HCV polyprotein spans the ER membrane, and all mature viral proteins are associated with the ER, mostly directly via several types of membrane anchors, and in the case of NS3 also involving the peripheral association to its NS4A cofactor [42]. At least two of these mature proteins, core and NS5A, are commonly believed to translocate to the lipid droplet surface [30,31].

Core protein is 21 kDa in size, and binds the LD monolayer after maturation of its precursor (23 kDa) by cleavage of its transmembrane anchor by the signal peptidase and the signal peptide peptidase [42]. This leaves the so-called domain 2 at the C-terminal extremity free to bind the lipid droplet surface [43,44] thanks to its two amphipathic α-helices (residues 119–136 and 148–164) separated by a hydrophobic loop [45] (Figure 2). Importantly, core domain 2 is sufficient to target the fused GFP to the lipid droplet surface [45]. HCV core shares together with GBV-B (GB virus B) core a number of features also described on plant oleosins: in addition to the hydrophobicity of the LD-binding domain, a proline knot in the hydrophobic loop and a conserved YATG (WATG in GBV-B core) sequence (required for core association with lipid droplets) are found on all three proteins [46]. Importantly, mutation of the two proline residues of the proline knot prevents core coating of the lipid droplets [32,46]. Therefore, core strongly resembles the prototype class I proteins oleosins [46].

NS5A is a 56–58 kDa phosphoprotein composed of three domains (DI-III). Its N-terminal extremity (residues 1–30) is responsible for membrane association, as attested by the targeting of GFP fusion constructs [47]. Circular dichroism established that the corresponding peptide folds as an amphipathic α-helix. This helix is predicted to form an in-plane membrane anchor, with a tryptophan-rich hydrophobic side towards the membrane and a charged hydrophilic side towards the cytosol [47] (Figure 2). Interestingly, these features are conserved among other members of the hepacivirus and pestivirus genera [48]. The helix strongly resembles the N-terminal lipid droplet anchor of the antiviral viperin protein and can direct viperin or the fused dsRed fluorescent protein to the lipid droplet surface [49]. Of note, NS5A interacts with several lipid droplet-associated proteins such as the PAT-protein TIP47 [50] or Rab18 [51] but also HCV core [52,53,54]. It is possible that these interactions facilitate NS5A binding to lipid droplets.

Although NS3 and NS4B have also been detected at the LD periphery, these proteins are believed to remain anchored within the ER membrane and only closely appose to the LD surface [30,31]. The NS4B topology is not fully clear but it harbors at least four transmembrane segments, which makes it an integral ER membrane protein [55]. Nevertheless, when expressed alone, a small portion of tagged NS4B forms rings around lipid droplets [56]. Actually, the N- and C-termini of NS4B can target a fluorescent protein to the LD surface. Each of these sequences might form an amphipathic α-helix responsible for the LD apposition (Figure 2). The authors therefore proposed that NS4B tethers ER and LDs [56].

### 3.2. Transfer of HCV Proteins from the ER to the LD Surface

#### 3.2.1. The Physiological Case

**Figure 3:**
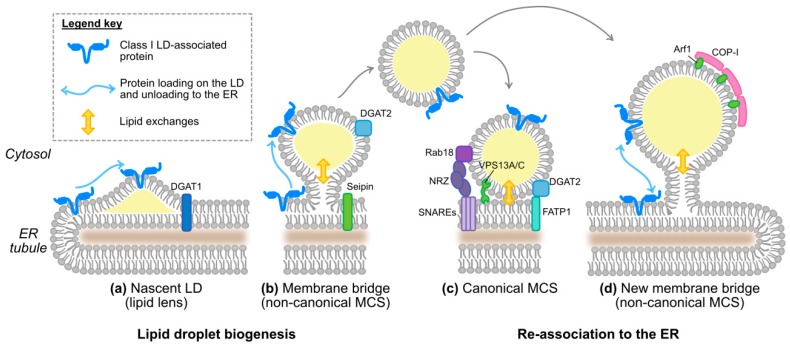
Contacts and exchanges between lipid droplets and the endoplasmic reticulum (ER). (**a**) Lipid droplets form by acyl-CoA:diacylglycerol O-acyltransferase 1 (DGAT1)-mediated triglyceride accumulation between the two ER leaflets. Some ER-associated proteins, such as ASCL3, a class I LD-associated protein, traffic to LDs during this early stage of LD production [59]. (**b**) Upon LD expansion catalyzed by DGAT2, some LDs maintain membrane bridges with the ER, in a process regulated by seipin [60]. (**c**) Detached lipid droplets might reconnect with the ER via conventional MCSs tethered by protein complexes (e.g., DGAT2-FATP1 [61], SNAREs-NRZ-Rab18 [62,63]) and/or by lipid transporters docked both on the ER and the LD surface (e.g., VPS13A/C) [64] (see main text). (**d**) Alternatively, new membrane bridges can reversibly reconnect LDs and ER and permit the regulated loading of the lipogenic protein GPAT4 [39] and the lipolytic protein ATGL [65,66] (not shown in the figure) from the ER. Membrane bridge formation involves the COP-I/Arf1 machinery. COP-I/Arf1 proteins are believed to extract phospholipids from the lipid droplet surface by triggering the budding of nano-lipid droplets, which have a very high surface to volume ratio. The phospholipid depletion results in an increased surface tension and lipid droplet instability, which promotes the establishment of membrane bridges with the ER [67]. Of note, the connections between LDs and ER have been investigated in various experimental systems (e.g., Drosophila S2 cells, human adipocyte cell lines, etc.) but their relevance in human hepatocytes or hepatoma cell lines often requires confirmation.

As lipid droplet-associated proteins have different origins (ER vs. cytosol), distinct mechanisms ensure their targeting to the lipid droplet [36]. Class II lipid droplet proteins are translated in the cytosol and directly shuttle to the lipid droplet. How they recognize this surface from the other intracellular membranes is not yet understood. On the contrary, the ER-derived class I lipid droplet proteins can translocate either during the formation of initial lipid droplets or after reconnection of expanding lipid droplets to the ER via membrane bridges (Figure 3a,b,d). Here, we will focus on this protein category to which HCV proteins belong.

Membrane bridges are non-canonical ER-to-LD membrane contact sites (MCSs) [60]. As opposed to the canonical MCSs, bridged organelles share a continuous membrane, in this case, the ER bilayer and the lipid droplet monolayer, a situation conducive to protein exchanges. Membrane bridges between ER and LDs first occur in a process involving Seipin [60,68,69] during lipid droplet biogenesis, when nascent lipid droplets emerge and expand from the ER membrane [1]. That is the time when some class I proteins such as ASCL3 (acyl-CoA synthetase long-chain family member 3) translocate onto lipid droplets. ASCL3 concentrates in ER microdomains, the lipid droplet nucleation sites, and already coats nascent lipid droplets even before their core lipid mass can be detected by conventional neutral lipid dyes and microscopy [59]. After biogenesis, some lipid droplets remain attached to the ER while others dissociate, in a reversible process depending on the coatomer COP-I/Arf1 machinery [39]. Membrane bridge reestablishment via COP-I/Arf1 could be unique to lipid droplets and is important for the transfer of drosophila GPAT4 [39] and of the ATGL (adipose triglyceride lipase) lipase [65,66] to the lipid droplet surface. The plasticity of membrane bridges between ER and LDs permits a membrane continuity between a lipid bilayer and a monolayer and might substitute for the vesicular transport of proteins that ensures continuity between two bilayers and protein transfer between other organelles, for instance along the secretory pathway. However, how proteins accumulate on the lipid droplet side of the bridge rather than equilibrating between the connected organelles remains elusive [36]. Note that conventional MCSs also exist between ER and lipid droplets [60] (Figure 3c) and will be discussed in another context (Section 4.2), as their participation in HCV protein translocation is unlikely, in absence of membrane continuity.

#### 3.2.2. HCV Case: Dynamics of HCV Hijacking of the Lipid Droplet Organelle

**Figure 4:**
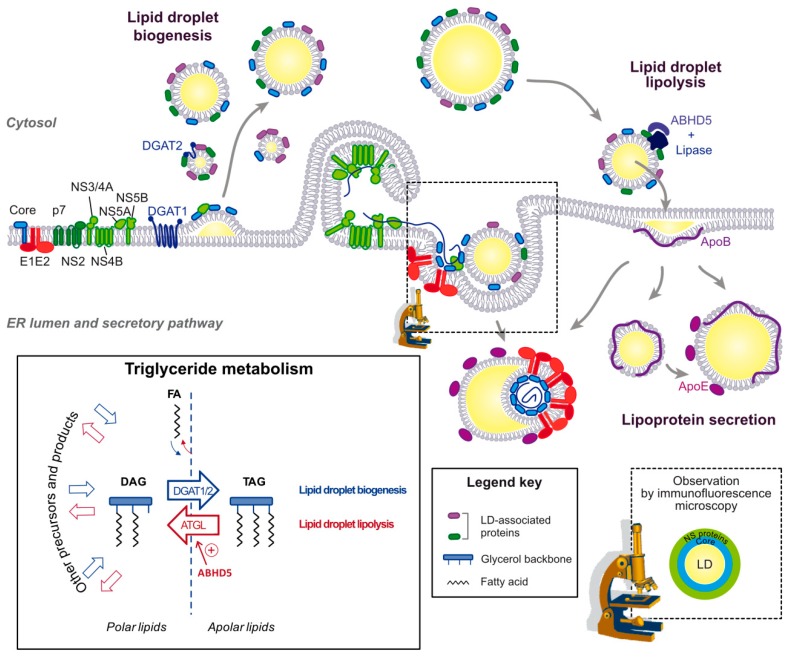
Model for HCV assembly and release. HCV morphogenesis likely occurs at contact sites between the ER-embedded replication machinery and envelope glycoproteins on one side, and the core-coated lipid droplets on the other side. This localization of the assembly complexes might facilitate the access to the lipoprotein pathway, with the incorporation of apolipoproteins and an excess of neutral lipids into nascent lipo-viral particles. A few host factors involved in HCV assembly are represented. Note that it is unclear how virus and lipoprotein morphogenesis converge. Please refer to the main text and references for discussion on this topic. The bottom left box illustrates the only committed steps in the triglyceride metabolism: the switch between diacylglycerol (DAG) and triacylglycerol (TAG), representing the last step in TAG synthesis and the first step in TAG lipolysis, respectively. Switching from DAG to TAG corresponds to a change in lipid polarity: the TAGs are apolar and excluded from membranes, therefore their accumulation promotes lipid droplet biogenesis, while the DAGs are polar and serve as intermediates to draw triglycerides from the lipid droplet inner core during lipolysis. We recommend the interested reader to refer to the review by Pol A. et al. for a comprehensive overview of the triglyceride metabolism [73].

Core seems to be the first HCV protein translocated from the ER to the lipid droplet surface, since in cells infected with the above-mentioned core double proline mutant virus (Section 3.1.2) not only core but also NS5A no longer traffic to the surface of LDs [31]. By confocal microscopy, NS5A and other NS proteins are found in a donut-shape around the core-coated lipid droplets [30,31] (Figure 4, bottom right inset). Interestingly, mutants of NS5A that fail to accumulate on the lipid droplets even in the presence of core, prevent the recruitment of other NS proteins to the LD surface [31]. Thus, this series of mutants reveals a stepwise co-opting of the lipid droplet by HCV proteins, first with the translocation of core, then the core-facilitated NS5A transfer and finally the NS5A-mediated apposition of the replication complexes that are embedded in ER-derived membranes [6]. This coordinated process requires not only the lipid droplet-targeting sequences encoded by HCV core and NS5A, but also concerted interactions between viral proteins and the participation of several host factors.

The initial hijacking of lipid droplets by core is likely to occur during lipid droplet biogenesis [70] (Figure 4). Rather than passive diffusion, it is DGAT1 that interacts with core and drags it onto the nascent lipid droplet, both in core-expressing and HCV-infected cells. Indeed, DGAT1 expression and enzymatic activity are both required for HCV core lipid droplet loading and for HCV assembly. Moreover, DGAT1 interacts with both the mature 21 kDa and unprocessed 23 kDa forms of core [70]. DGAT1 further interacts with NS5A and reinforces the NS5A-core interaction in a tripartite complex [54]. A mutant of DGAT1 was described to inhibit in a dominant negative manner NS5A translocation to the lipid droplet surface without affecting core localization, suggesting that DGAT1 also has a role in NS5A lipid droplet localization that is independent of core [54]. Importantly, there are two enzymes responsible for the last and only committed step in the triglyceride synthesis: DGAT1 and DGAT2 [71]. Despite their similar enzymatic function, the proteins belong to distinct families and share no sequence or structural homology [71]. Furthermore, DGAT1 resides in the ER while DGAT2 also traffics to the lipid droplet surface [1]. Functionally, DGAT1 and 2 were proposed to catalyze triglyceride synthesis destined for distinct lipid droplet pools: the initial lipid droplets for DGAT1 and the subset of expanding lipid droplets for DGAT2 [1] (Figure 3a,b). So far only DGAT1 has been involved in HCV replication cycle, and its interaction with core or NS5A or its participation in HCV assembly is not reproduced by DGAT2 [70]. Possibly, HCV has evolved a mechanism to use a specific lipid droplet subset: those derived from DGAT1- but not the ones from DGAT2-triglyceride synthesis. The benefits to the virus might come from a functional speciation of these two lipid droplet subsets. Alternatively, DGAT1 is simply the only lipid droplet entry door that is successfully opened by the virus and the dual interaction of DGAT1 with core and NS5A ensures that the complete HCV machinery is addressed to the same lipid droplet subset. Of note, the cytosolic calcium-dependent PLA2G4A phospholipase is also implicated in core recruitment to the lipid droplet, and its pyrrolidine-2 inhibitor potently impairs HCV assembly by targeting multiple steps of the virion morphogenesis [72]. Also, apart from DGAT1, several other LD-associated proteins, including Rab18 and TIP47 can interact with NS5A and might participate in recruiting this protein to the lipid droplet [50,51].

## 4. Lipid Droplets as an Entry Door for HCV to Reach the Site of Lipoprotein Biogenesis

### 4.1. Lipid Droplets Are a Stopover during the Assembly Process

While HCV capsid protein accumulates at the lipid droplet surface early after its processing, the viral envelope glycoproteins are ER-resident [30] and their glycosylation pattern at the virion surface attests the virion trafficking through the secretory pathway [74]. The block in HCV particle secretion by Brefeldin A, an inhibitor of the vesicular transport between ER and Golgi [75], further supports the notion that HCV buds into an early secretory compartment, presumably the ER. This implies that core is recruited back from the LD to the ER membrane for particle production and budding.

Importantly, a switch between ER and LD localization has also been described for some class I lipid droplet-associated proteins, although the unloading from the LD to the ER is not well understood [36]. Typically, class I proteins are often removed for degradation, either directly at the lipid droplet surface, or after re-localization to the ER and through involvement of the ER-associated degradation (ERAD) pathway. Contrary to class II proteins that can be unloaded by simple molecular crowding, for instance during lipid droplet shrinking by lipolysis, class I proteins likely require dedicated machinery, as their hairpin is deeply embedded in the phospholipid monolayer and contacts the LD hydrophobic core. Notable, the lipid droplet metabolic status (expansion vs. shrinking) correlates with the shuttling of some proteins between lipid droplets and ER [76]. As lipolysis plays an important part in HCV assembly [77], it would be interesting to determine whether virus budding occurs close to shrinking lipid droplets and whether this shrinking can favor the unloading of core from the lipid droplets to the budding site at the ER membrane. In the case of HCV, this retrieval of core might coincide with changes in the protein properties, possibly triggered by viral RNA binding, oligomerization and/or capsid formation. However, presently it is not clear whether the returned core protein actually interacts directly with the ER membrane or whether its phospholipid association domain is masked by conformational changes.

Evidence for core unloading from the lipid droplet organelle was obtained indirectly by the comparison of lipid droplet loading of different HCV strains [78,79], by arresting virus assembly (e.g., [80,81,82]) and by imaging the trafficking of tetracysteine-tagged core in live infected cells [80]. Thus, using a dual labeling pulse-chase approach and a tetracysteine-tagged core HCV variant, Counihan and colleagues could follow the fate of two pools of core proteins synthetized at different time points [80]. Core protein was translocated to the lipid droplet organelle shortly after synthesis and this loading lasted at least several hours until the number of lipid droplets coated by core decreased and more LD-independent core puncta were observed, possibly reflecting virion-transporting vesicles [80]. Secondly, arrested virus assembly often correlates with the exaggerated accumulation of core at the lipid droplet surface. This has been reported multiple times, with virus mutants blocked in assembly [80,81], assembly inhibitor treatment (e.g., Brefeldin A [80]), or in cell lines lacking important HCV assembly cofactors (e.g., ApoB/ApoE knockout hepatoma cell lines [82]). Along the same lines, core protein of the JFH1 strain accumulates readily at the lipid droplet surface. However, this lipid droplet association is much decreased for closely related strains that are more efficient in HCV assembly, for instance, a cell-culture adapted version of JFH1 or the chimeric Jc1 virus, a chimera between JFH1 and the related J6 genome [78,79]. These differences could be traced back to changes in core protein [78] or p7 and NS2 sequences [79]. Indeed, bringing together the genome, capsid and envelope proteins of HCV requires the concerted action of its structural and non-structural proteins. This process is mostly orchestrated by NS2 and p7 and has been extensively reviewed elsewhere (e.g., [83]). Other viral proteins also influence core recruitment back to the ER. As a recently published example, an NS3 adaptive mutation in HCV JFH1 strain increases core reticular localization, capsid envelopment and results in a 10-fold increase in infectious titer [84].

Altogether, these cumulated pieces of evidence indicate that the accumulation of core at the lipid droplet surface is an essential yet intermediate step in HCV assembly. The site of particle assembly and ultimately budding seems to be the ER. This mechanism that involves spreading of viral structural proteins onto two distinct organelles and the necessary dynamics to gather these elements implies well-tuned connections between the ER and the lipid droplets, as detailed in the next section.

### 4.2. The Interface between LDs and ER as an Assembly Platform

It is likely that the physical proximity between core-loaded lipid droplets and ER-resident glycoproteins and replication complexes is a requirement for the unloading of core, the initiation of virion assembly, the budding and the maturation of the viral particles. The assembly platform indeed straddles two organelles, lipid droplets and ER, which maintain close and regulated connections. Recently, the concept of membrane contact sites has emerged as a new and integrative type of inter-organelle interactions [60]. It is conceivable that HCV promotes such membrane contact sites to keep together its assembly platform. Note that for simplicity, the term “ER” covers here both the conventional ER as well as the virus-modified ER membranes, also called membranous web, that hosts the replication complexes within double-membrane vesicles [6].

First of all, it is plausible that some core-loaded lipid droplets do not detach from the ER membrane and that HCV assembly machinery gathers at the junction between the continuous ER bilayer and the lipid droplet monolayer, across the previously mentioned membrane bridges (Figure 3b). Lipid droplets that are docked to the ER can remain highly mobile and circulate along the ER tubules [69], which could even increase chances to form assembly sites.

Of note, core protein is able to displace ADRP from the lipid droplet surface, which results in a microtubule- and dynein-driven lipid droplet clustering towards the microtubule organizing center (MTOC), in the perinuclear area and close to HCV replication sites [85]. Although the phenotype was blurred with highly assembly-competent viruses probably due to the high core turnover [78], lipid droplet concentration close to the nucleus was confirmed in JFH1-infected cells [78,85]. Lipid droplet clustering is facilitated by HCV upregulation of Septin 9 and the increased formation of septin filaments [86]. This mechanism could facilitate the proximity between core-loaded and ER-detached lipid droplets and ER-resident viral proteins.

The pathways of membrane bridge formation [67] could be used for the docking of HCV-aggregated perinuclear lipid droplets to the ER (see above, Section 3.2.1 and Figure 3d). However, like other organelles, lipid droplets are also able to form conventional MCSs with the ER [60]. In such contact sites, the lipid droplet hemilayer is opposed to the ER bilayer, without fusion. This setting might not permit protein shuttling between the organelles as membrane bridges do, but can promote lipid exchanges and form a physical tether between lipid droplets and ER tubules (Figure 3c).

In the HCV field, attention was drawn towards the MCS via the mitochondria-ER contact site with the discovery that the MAMs (mitochondria-associated ER membranes) coordinate the MAVS (mitochondrial antiviral-signaling protein)-mediated antiviral innate immune response against HCV [87]. Furthermore, the reorganization of PI4P (phosphatidylinositol 4-phosphate) lipids in HCV infection has further captured the interest for ER contact sites and the lipid transporters regulating the membranous web formation [88]. For instance, OSBP (oxysterol binding protein) and CERT (ceramide transfer protein) shuttle lipids between organelles and have been shown to facilitate HCV infection [89,90]. These proteins bind on one hand Golgi lipids and on the other hand the ER-anchored VAP proteins (vesicle-associated membrane protein-associated proteins) via the lipid transporter FFAT motif (two phenylalanines in an acidic tract) [60]. This dual docking stabilizes the contact between the two organelles and allows lipid exchanges along or against their concentration gradients [91]. VAPs also regulate ER MCSs with endosomes, peroxisomes or the plasma membrane [60].

Tethering complexes and lipid transporters have also been reported at the ER-LD junction (Figure 3c). For instance, VPS13A and C are glycerolipid transporters localized at ER-LD MCSs with two functional heads: a predicted FFAT motif for interaction with ER-resident VAP proteins in the N-terminal moiety and a lipid droplet binding site in the C-terminus including a predicted amphipathic α-helix [64]. Furthermore, two protein complexes have been identified that mediate MCSs between ER and LDs [60] (Figure 3c). The first complex involves the ER-resident FATP1 acyl-CoA synthetase and the LD-associated DGAT2 [61]. The second one bridges ER-resident SNAREs (soluble N-ethylmaleimide-sensitive factor attachment protein receptors) with LD-associated Rab18 via the ER-associated NRZ complex (NAG-RINT1-ZW10) [62,63]. Interestingly, Rab18 plays a role in HCV replication cycle, possibly in replication and/or assembly [51,92]. Consistent with its physiological tethering role, Rab18 interacts with HCV NS5A and promotes the co-localization between viral replication complexes and lipid droplets [51]. Curiously however, in live infected cells, tetracysteine-tagged core was excluded from Rab18-positive lipid droplets [80]. This might reflect efficient back-transfer of core to the ER from LDs that are tethered to the ER through a Rab18-coordinated MCS. Another host protein suggested to bridge HCV-usurped lipid droplets and replication complexes is TIP47 (tail interacting protein of 47 kDa). TIP47 is a PAT-protein and as Rab18, it interacts with NS5A, and has been implicated in mediating HCV replication and/or assembly [50,93]. Finally, the HCV NS4B protein was also proposed as an ER-LD tether, with several transmembrane segments spanning the ER membrane and one amphipathic α-helix on each side apposed in plane to the lipid droplet surface [56] (see Section 3.1.2 and Figure 2). Of note, using a dual fluorescent labelling pulse-chase, Wang and colleagues observed that cholesterol enrichment of previously assembled HCV replication organelles was a prerequisite for the apposition of replication complexes and lipid droplets, which only started 16 h after the replication complex formation [94].

Presumably, the benefit of this virus “pit stop” at the lipid droplet is to recruit this essential metabolic hub to the assembly factory. The functional consequences seem to be dual: (i) access to the lipid droplet neutral lipid stores for the lipidation of the lipo-viro-particle, (ii) usurpation of part of the connected lipoprotein assembly pathway and incorporation of host apolipoproteins. These aspects are discussed in the following two sections.

### 4.3. Importance of Triglyceride Hydrolysis for HCV Morphogenesis

Lipid droplets are the main source of triglycerides exported in the VLDL and likely for HCV lipo-viro-particle. The apolar nature of the triglycerides precludes their incorporation in membranes. Triglyceride circulation between cell compartments, therefore, depends on a cycle of hydrolysis and re-esterification [95]. Like the synthesis of the triglycerides, their hydrolysis is a multistep reaction whereby the three fatty acids are sequentially removed. In adipocytes, ATGL catalyzes the first and only committed step, it is activated by ABHD5 (α/β hydrolase domain-containing protein 5, also known as CGI-58) and repressed by G0S2 (G0/G1 switch gene 2) [96,97] (Figure 4, bottom left box). Note that mutations in both, ATGL and its cofactor ABHD5, are associated with neutral storage diseases in human [98]. Removal of the second and third fatty acids involve the rate-limiting hormone-sensitive lipase (HSL) and the monoglyceride lipase (MGL), respectively [73].

A number of intracellular lipases have been identified as cofactors for HCV morphogenesis but most were phospholipases and their proviral effect could not be tracked down to a clear link with lipid droplet lipolysis [72,99,100,101]. Interestingly, AADAC (arylacetamide deacetylase), a putative triglyceride lipase involved in VLDL secretion, was found to be involved in HCV replication cycle and to be downregulated in vitro in HCV-infected cells. AADAC knockdown dramatically decreases infectious virus production but several steps of the viral replication cycle seem to be influenced [102]. Moreover, we identified the ATGL cofactor, ABHD5/CGI-58, as a host factor promoting HCV assembly and release [77]. ABHD5 (α/β hydrolase domain-containing protein 5) is a lipid droplet-associated protein, although it is not clear whether it belongs to the class I or II [36]. Its lipid droplet association is conferred by a predicted α-helix in its N-terminus and by the enclosed tryptophan stretch [103,104]. We pinpointed the lipase cofactor activity as the proviral function. In particular, human variants of ABHD5 responsible for the Chanarin-Dorfman neutral lipid storage disease are unable to degrade the lipid droplets and to support HCV production in liver-derived cells [77]. Although the phenotypes were too mild and complex to determine the virion lipidome after ABHD5 perturbation, our study supported a role for the neutral lipid transfer between cytosolic lipid droplets and the ER membrane in HCV morphogenesis. We are currently working on the identification of the lipase activated by ABHD5 and responsible for its proviral effect. Indeed, there has been some controversy on whether ATGL is an ABHD5 effector in the liver or not: (i) ATGL expression in the liver has been debated, (ii) liver dysfunctions are more commonly associated with ABHD5 rather than ATGL deficiencies, whether in mouse models [105] or in human [98], and (iii) there are indications for ATGL-independent functions of ABHD5 in the liver (e.g., ABHD5 regulates mouse liver inflammation and TAG metabolism even in ATGL knockout animals [106]). Moreover, although ATGL is the main player in the adipocyte lipolysis and is a functional lipase in the murine liver [107,108], it only accounts for less than half the hepatic TAG hydrolase activity in fasted mice and its contribution to VLDL biogenesis is contested [109]. Identification of the ABHD5-activated lipase involved in HCV assembly will complete one piece of the puzzle to understand how the HCV lipo-viro-particle acquires its neutral lipid core. The ER enzymes catalyzing the re-esterification of the triglycerides and the whole fate of the cholesteryl esters, also incorporated in the virion but handled in the cell by completely different machinery [73], will still need to be identified.

Regarding the triglyceride re-esterification, it is noteworthy that the TAG synthesis enzyme DGAT1 was proposed to have a dual membrane topology at the ER membrane compatible with both a latent (luminal) and an overt (cytosolic) activity [71,110]. The luminal activity might participate in lipoprotein genesis [71]. DGAT2 topology, on the contrary, is only compatible with a cytosolic activity [111]. While so far only the overt activity was directly involved in HCV production [54,70], this intriguing DGAT1 property might be co-opted by HCV to enter the lipoprotein production pathway and re-esterify the triglycerides for their incorporation in the lipo-viro-particle. Using the same enzyme to hijack the lipid droplet organelle and to load the virion lipid cargo would concentrate the assembly machinery and increase its efficiency.

Finally, TM6SF2 is important for a post-assembly step in HCV production [112]. TM6SF2 is mostly expressed in the liver and the intestine [113] and was first identified in an exome-wide association study as a determinant for nonalcoholic fatty liver disease [114]. In mice, TM6SF2 knockout results in liver steatosis, and congruently with decreased plasma triglycerides, but intact ApoB secretion. In fact, while lipid droplets and their neutral lipids accumulate, the secreted VLDL are smaller and poorly lipidated [113]. Consistently, TM6SF2 facilitated the secretion and subtly influenced the density of HCV lipo-viro-particle, supporting the importance of the lipid droplet as a source of neutral lipids for the virion [112]. Unfortunately, the precise function exerted by TM6SF2 in VLDL lipidation is unknown, and in particular, it is still unclear whether an enzymatic activity or a lipid transport function is involved.

### 4.4. Key Role of Apolipoproteins in HCV Production in Liver Cells

The incorporation of several apolipoproteins in the HCV particle lead to the identification of ApoE, ApoB and ApoC-I, but also of MTTP (microsomal triglyceride transfer protein) and CIDEB (cell-death-inducing DFFA-like effector B) as possible host factors for HCV morphogenesis [9,115,116]. The relative contribution of each of these factors, in particular of the apolipoproteins, in HCV assembly, however, remained controversial [9,115]. These difficulties are to be attributed to several obstacles. First of all, lipoprotein production is still a poorly understood process where genetic or pharmaceutical intervention on an individual factor can induce a more global response, leading to false interpretations. As an example, the antiviral effect of MTTP inhibitors was later linked to a decrease in ApoE secretion [26]. Secondly, the lack of an ectopic expression system has impeded studies on the role of ApoB, the largest apolipoprotein (550 kDa). Thirdly, the typical hepatoma cell lines used in HCV research do not fully mimic the lipoprotein synthesis pathway from primary hepatocytes. HCV-permissive cells typically express a wide range of apolipoproteins, whose relative expression varies between hepatoma cell lines used and differs from primary human hepatocytes or the in vivo situation [82,117]. It was assumed for instance that ApoB was relevant for HCV morphogenesis in vivo and in primary hepatocytes but dispensable in cultured hepatoma cell lines [115]. Along the same line, both the Huh-7 and HepG2 cell lines only produce poorly lipidated ApoB-containing lipoproteins [10,115,118]. In contrast, induced pluripotent stem cell-derived hepatocyte-like cells (HLCs) are able to secrete VLDL-like lipoproteins [119] and are permissive to HCV infection [120]. Unfortunately, technical challenges, high costs and rather low virus production in this system hinder the generalization of the model, which could otherwise become a new gold standard to validate the role of the lipoprotein machinery in HCV production.

**Figure 5:**
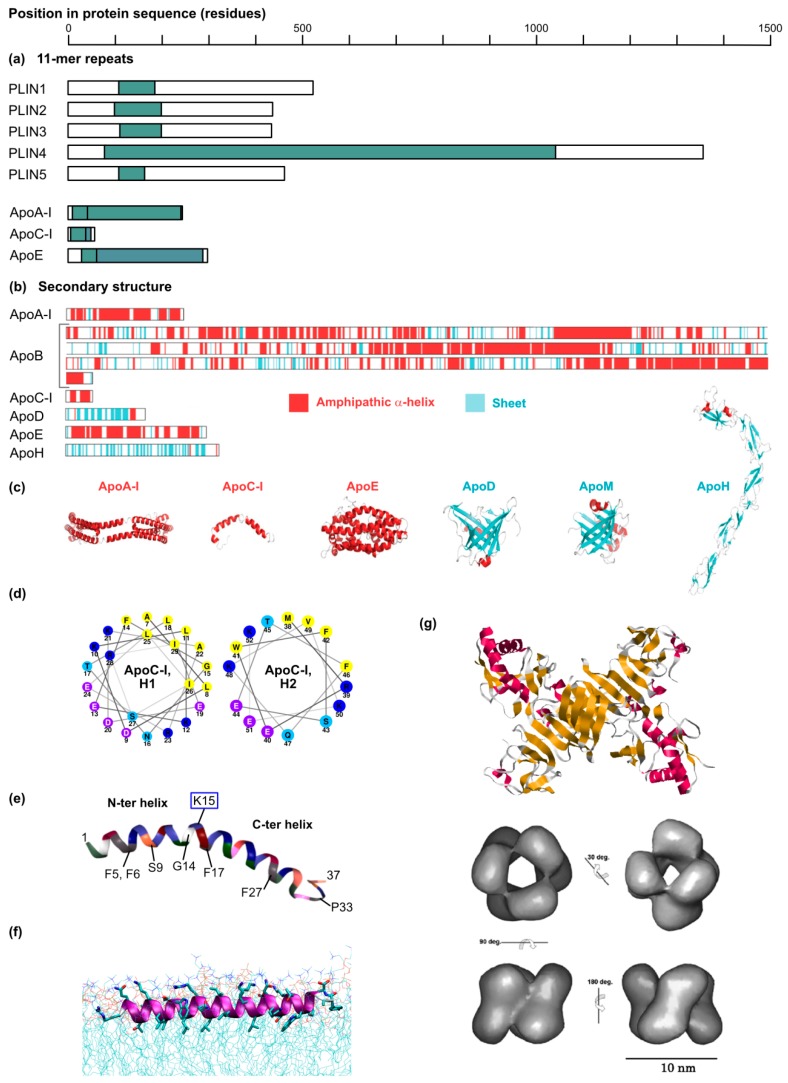
Striking sequence and structural features of apolipoproteins and apolipoprotein mimics. (**a**) Like the perilipins, apolipoproteins contain 11-mer repeats (teal-colored boxes) that have the propensity to fold as amphipathic α-helices. ApoA-I, C-I and E are shown as examples. For the apolipoproteins (the mature proteins are depicted), the two colored boxed correspond to 11-mer repeats found in exons 3 and 4, respectively. In some cases, the 11-mer repeats are organized themselves into larger motifs: 33-mer (3 × 11-mer) repeats for PLIN4 [154], 22-mer repeats for most 11-mers found in exon 4 of the apolipoproteins [155]. Note that PLIN2 is also called ADRP, and PLIN3 corresponds to TIP47. The coordinates of the 11-mer-containing domains have been extracted from Copic A. et al. [154] for the PLIN proteins and from Luo C.C. et al. [155] for the apolipoproteins. (**b**,**c**) Predicted or experimentally determined secondary structures and 3D models of a range of apolipoproteins. Note that ApoA-I, ApoB, ApoC-I and ApoE, which are predominantly folded as amphipathic α-helices, can rescue HCV virus production in 293T-derived cells but ApoD and H, which are mostly organized in sheets, cannot [82,117]. These two panels were reproduced and adapted from Fukuhara T. et al. [82] with permission of the authors. (**d**) Wheel representations of the two ApoC-I α-helices. The helices were drawn with Netwheels (http://lbqp.unb.br/NetWheels/) [58] and the color coding is as in Figure 2. (**e**) Structure of CAMP LL-37 domain in lipid micelles as determined by NMR (PDB ID: 2K6O, [139]) and visualized with RCSB PDB (www.rcsb.org) [156] and the NGL Viewer [157]. Key residues of the hydrophobic face apposed to the lipid micelles are indicated on the bottom. The lysine residue responsible for the hinge between the N- and C-terminal helices is indicated on the top and highlighted with a blue square. (**f**) E^rns^ amphipathic α-helix (residues 194–227) reconstituted by molecular dynamics in a theoretical membrane. Reproduced from Aberle D. et al. [146] with permission of the authors. (**g**) Top panel: Crystal structure of the NS1 protein dimer of dengue virus type 2. Note the predominant sheet organization. Structure published in Akey D.L. et al. [149], PBD ID = 4O6B, visualized with Rastop, a software adapted from Rasmol (http://www.geneinfinity.org/rastop/) [158]. Bottom panel: 3D reconstruction of dengue virus 1 NS1 hexamer based on cryo-electron microscopy. The hexamer forms an open barrel of about 10 nm in diameter with a central channel (see top right image) believed to accommodate the neutral lipid core of this HDL-like particle. Reproduced from Gutsche et al. [151] with permission from the authors.

In the last years the incremental comprehension of HCV tissue tropism and technological advances such as the popularization of gene knockouts have shed new light on the role of the lipoprotein synthesis pathway in HCV production, with three major findings: (i) Strikingly, ectopic ApoE expression efficiently complements the HCV production machinery in non-hepatoma cell lines (e.g., in the HEK 293T or HeLa cell backgrounds) that are otherwise incapable of producing infectious particles [121,122]. Importantly, these non-hepatoma cell lines are quasi devoid of the lipoprotein synthesis equipment as they only express minute amounts of MTTP, CIDEB, ApoB or other apolipoproteins [122,123]. Thus, although CIDEB might interact with HCV NS5A and facilitate ApoE incorporation in the virion [116], it is overall dispensable for HCV production. Accordingly, it was deduced that ApoE is the key lipoprotein production factor involved in HCV morphogenesis. (ii) Apolipoproteins have redundant roles in HCV production. In the non-hepatoma cell lines mentioned above, actually all ApoE-related exchangeable apolipoproteins [82,117] but also ApoB [82] are capable of restoring HCV production. Consistently, knocking out the expression of both ApoB and ApoE dramatically reduced HCV production in hepatoma cell lines, while individual knockouts had only mild phenotypes [82]. Using this system, the role of MTTP was pinpointed to ensuring the proper ApoB maturation, and therefore MTTP was only critical for HCV infection when ApoE was absent [82]. (iii) Rather than a particular amino acid sequence, it is the amphipathic α-helical structure, shared by all ApoE-related apolipoproteins as a series of conserved 11-mer repeats and present in ApoB, that is the key determinant for HCV assembly [82,117] (see Section 8 below) (Figure 5a–d).

## 5. Plasticity and Maturation of HCV Lipo-Viro-Particle

The resemblance between HCV virion and lipoproteins is not simply physical but also functional. Lipoproteins categories, VLDL, LDL, IDL and HDL (respectively very low, low, intermediate and high density lipoproteins) are dynamic populations that exchange lipids and apolipoproteins [124]. Lipid transfer or recruitment is catalyzed by serum enzymes, for instance, CERT (cholesterol ester transfer protein) transfers cholesterol from HDL to VLDL, and lipoprotein lipase, which is anchored to the vascular surface, hydrolyzes triglycerides from the circulating VLDL to provide fatty acids to underlying cells [125]. Furthermore, apart from ApoB, apolipoproteins reversibly adsorb to the lipoprotein surface and can switch between the lipoprotein classes within the circulation. The reversible change between lipoprotein-bound and lipid-free states is accompanied by a dramatic structural reorganization of the apolipoprotein, as illustrated for ApoE [126,127].

Similarly, HCV virion density range is not only broad but also dynamic. In the bloodstream, HCV shifts to a lower density after a high-fat meal, suggesting an intravascular transfer of lipids between lipoproteins and lipo-viro-particles [11]. Consistently, in human liver chimeric mice, induction of lipoprotein secretion and lipidation by providing a high-sucrose diet had a very modest effect on the virus density profile [128]. Virion plasticity and lipid enrichment was also observed in vitro where the HCV density profile from the plasma of a fasted infected individual was shifted after supplementation of a lipid emulsion or of plasma from a naive patient in postprandial state [11].

On the other hand, the apolipoprotein coat of HCV is also not rigid. This can be strikingly evidenced with HCVcc produced in conventional cell culture systems. ApoE content in the supernatant of Huh-7-derived cells is about 100-fold lower than in human plasma [129]. In these conditions, secreted virions can be loaded with additional ApoE, by supplementing the viral supernatant with cell supernatant from hepatoma [129,130] or non-hepatoma cells [131] overexpressing ApoE. Commercial recombinant non-lipidated ApoE can also be incorporated into the virions, although much less efficiently [130]. ApoE stoichiometry on plasma-derived HCV exceeds by far the decoration harbored in the HCVcc system, nevertheless, even genuine virions from human sera can load exogenous ApoE [129], suggesting that ApoE exchange can occur in the bloodstream. Interestingly, ApoE exogenous complementation increases virion infectivity by boosting entry [129,130] and furthermore dramatically decreases the virion sensitivity to antibody-mediated neutralization [129]. Extracellular loading of ApoC-I on the viral particle has also been observed [132,133,134]. Unlike ApoE, ApoC-I incorporation involves the lipid receptor SR-BI and HCV E2 hypervariable region 1 [129,132,133,134]. However, functionally, ApoC-I supplementation is at best mildly proviral, and upon high dose, clearly virocidal [134]. Whether other exchangeable apolipoproteins can also be acquired extracellularly is likely but will require further investigation.

## 6. Apolipoproteins: The Only Hitchhike for HCV to Exit? A Range of Proteins Can Substitute for Apolipoproteins in HCV Assembly and Egress

Comparison of the apolipoproteins supporting HCV production strongly indicates that structural and functional rather than sequence features are the critical proviral determinants and hints at the possibility that completely unrelated proteins or synthetic constructs might be able to substitute for the role of apolipoproteins in HCV assembly, egress and eventually entry. The Matsuura’s laboratory elegantly investigated this hypothesis by using the 293T or the double ApoE/ApoB knockout Huh-7 cell background. Among a small library of human secretory proteins containing α-helices, they identified human cathelicidin/cationic antimicrobial peptide (CAMP/hCAP18) as well as serum amyloid A4 (SAA4) as proteins able to compensate for the roles of apolipoproteins in HCV production [135]. Interestingly, SAA4 is an apolipoprotein that can exchange with Apo A-I and A-II on HDL particles [136], swap between lipoprotein categories [137] and inhibit HCV entry [138]. The authors focused on CAMP and confirmed again with this protein the crucial role played by the amphipathic α-helices [135], in this case, two juxtaposed and slightly tilted helices separating by an helix-breaker lysine residue and constituting the so-called LL-37 domain [139] (Figure 5e). CAMP has a number of interesting properties: it binds lipoproteins in plasma [140] and glycosaminoglycans [141] both via its C-terminal LL-37 domain [140,141]; moreover, it can bend membranes [142]. The idea that CAMP might be able to drive the secretion of lipoprotein-like particles is only a step away and might be worth investigating.

More recently, the same group published new compelling findings: the flaviviral NS1 protein and the pestiviral E^rns^ can both substitute for the role of apolipoproteins in HCV production, and vice versa ApoE and NS1 can at least partially reproduce the role of E^rns^ in pestivirus production [143]. At least for E^rns^, the amphipathic α-helices are instrumental to this function. NS1 and E^rns^ are secreted glycoproteins and accomplish several functions in the flaviviral and pestiviral replication cycles. Despite the common genome organization among the *Flaviviridae*, they are not encoded by the hepacivirus genus. In light of these findings, the authors propose that HCV has lost its secreted glycoprotein gene important for virus assembly and evolved to use, instead, the abundant apolipoproteins of the host hepatocytes [143].

Again, several features of NS1 and E^rns^ are calling for attention in the context of HCV assembly (Figure 5f,g). E^rns^ is a secreted 42–48 kDa protein that forms disulfide-linked homodimers [144]. Its C-terminus forms an atypically long amphipathic helix (up to 50 residues) that anchors it to lipid membranes [145] with a tilted orientation, so that its C-terminal end can insert in the membrane [146] (Figure 5f). Whether E^rns^ is secreted as a soluble homodimer or as part of a lipoprotein-like structure has to our knowledge not been investigated. However, it is worth mentioning that E^rns^ is present on the pestiviral particle and essential for pestiviral particle assembly [147] and that the virion buyoant density of pestiviruses (1.12–1.15 g/mL) is roughly compatible with an HDL-like organization (1.061–1.210 g/mL) [9]. Also, E^rns^ interacts with cell surface glycosaminoglycans via a cluster of basic residues [148] and is a target for mildly neutralizing antibodies [147] suggesting that it could also participate in the entry of artificial E^rns^-complemented HCV particles.

The 50-kDa flaviviral NS1 protein is a fascinating molecule. Its crystal structure shows a predominance of the beta-sheet fold with only two α-helices, whose possible amphipathic character is unclear [149] (Figure 5g, top panel). What is particularly interesting is NS1 dynamic interaction with lipids. First translated and released in the ER as a soluble monomer, it is able to homodimerize and consequently to attach to the ER membrane where it plays an important role in the virus replication machinery, but also to form hexamers that are secreted and involved in immune evasion [150]. Most interestingly, while the dimer partitions with the detergent in a detergent phase-partitioning assay, NS1 hexamers are recovered from the aqueous phase [151]. In fact, the 3D-reconstituted NS1 hexamer forms a 10-nm diameter barrel, symmetrically organized, with a wide central channel, as reconstituted from cryoelectron microscopy pictures [151] (Figure 5g, bottom panel). A combination of biochemical and modelling approaches predicted the incorporation of around 70 lipid molecules within the NS1 barrel, including on the one hand phospholipids, mono- and diacylglycerols, some cholesterol and sphingomyelin, all polar lipids that might belong to the particle surface, and on the other hand 6 molecules of triglycerides and 16–33 cholesterol esters, likely packed in the hydrophobic core of the structure [151]. In short, the lipid content of NS1 hexamers is strikingly reminiscent of HDL particles [151]. It is noteworthy that the aspect of HDL lipoproteins and NS1 hexamers differ: HDL particles have a large lipid cargo partly covered by the flexible and elongated ApoA-I molecules [152] whereas the denser NS1 hexamers form a thick and rigid symmetrical protein shell enclosing a small lipid core [151]. NS1 might recruit neutral lipids upon its dimer-to-hexamer transition [151]. Furthermore, NS1 secretion is sensitive to inhibitors of the lipid droplet (e.g., DGAT2 inhibition) and lipid raft metabolism (e.g., cholesterol sequestration), suggesting it really forms an atypical HDL particle [151], an organization that fully conforms to the newly resolved crystal structure [149]. Finally, like E^rns^, CAMP and ApoE, also NS1 binds cell surface glycosaminoglycans [153] so that it could participate in the attachment and entry of artificial *trans*-complemented HCV particles. It is odds-on that more proteins are able to substitute for apolipoproteins in HCV assembly, release and eventually entry.

## 7. Connections and Bifurcations between HCV and Lipoprotein Secretion Pathways

Despite the remarkable resemblances between HCV and VLDL particles, HCV morphogenesis does not phenocopy the VLDL biogenesis. As mentioned above, MTTP and ApoB are both crucial for VLDL secretion, they participate in the production of the primordial poorly lipidated VLDL particle [125,159], but both are dispensable for HCV production [82,121,122]. ApoE itself, which was seen as the key lipoprotein pathway player in HCV assembly, has been somewhat marginalized since it can be substituted by seemingly functionally unrelated proteins [135,143].

Even in liver-derived cells, where assembly depends on apolipoproteins, some doubt has been raised on whether virion and lipoproteins are produced in the same pathway. Microscopy evidence suggests the co-trafficking of ApoE and core in infected cells [28,160]. Backing this up, infectious virions, HCV structural elements (RNA, Core, envelope glycoproteins) and apolipoproteins were both found in immunoaffinity-purified COP-II vesicles that are in charge for cargo transfer between the ER and the Golgi [160]. Furthermore, a number of proteins or signaling lipids involved in the ER-to-Golgi or in the Golgi transport are important for HCV egress as well as in the VLDL secretion [28,89,160,161]. This includes OSBP, GOLPH3 and the PI4P lipid [89,161], but also CIDEB, KLHL12 or Sortilin [160]. Furthermore, several inhibitors block both HCV and lipoprotein secretions. This is the case for example of Brefeldin A, a broad inhibitor of the vesicular transport between ER and Golgi [75,162], of the more specific glycogen synthase kinase (GSK3)-β inhibitors, which otherwise leave albumin release intact [163], and of the lipid-lowering drug avasimibe which targets ACAT (acyl coenzyme A:cholesterol acyltransferase) [164]. Finally, the presence of complex glycans on HCV envelope glycoproteins attest their passage through the Golgi, while the many remaining high-mannose glycans indicate that not all glycosylation sites are accessible to the glycan-modifying enzymes, suggesting that the glycoproteins might travel through the Golgi as part of the virion-associated high-order covalent structures [74]. Altogether, these observations support the budding of HCV virions at the ER and their trafficking through the Golgi, just like lipoproteins.

Nevertheless, another body of evidence supports a VLDL-independent HCV egress involving endosomes, autophagosomes, exosomes or the ESCRT proteins [165,166,167,168]. Thus, a dominant negative variant of the Rab1b GTPase blocks the secretion of ApoB100, albumin and HCV, but not ApoE release [169]. More globally HCV and VLDL release differentially depends on a range of secretory Rab-GTPases and trans-Golgi adaptors [170] but also on clathrin and its AP-1 adaptor [165]. What is more, the ionophore Monensin A blocks VLDL but not HCV secretion [170]. Admittedly, different robustness in VLDL and HCV production and secretion, and pleiotropic effects of some cell factors in different steps of HCV replication cycle could account for some divergent responses to particular inhibitors. Yet, these discrepancies are striking and more perturbation studies, targeting a broad range of host factors with multiple levers, more relevant cell systems for both HCV and VLDL production and advanced high-resolution imaging methods are desirable to formally distinguish the routes taken by HCV and VLDL.

## 8. The Function of Apolipoproteins or Mimics in HCV Production—Hints and Guesses

A number of studies have tackled the role played by ApoE during HCV production, by analyzing HCV production in absence of ApoE [82,122,171], by pinpointing the apolipoprotein proviral domains or motifs [82,117], or by identifying viral ApoE interaction partners [9,171]. Thus, in the non-hepatic 293T cell line, proteinase K resistance assays suggest that virion envelopment proceeds in absence of ApoE, however neither intracellular nor extracellular infectious viruses are produced, indicating that ApoE is essential for a post-envelopment step of virus assembly [122]. These results were confirmed in hepatoma cell lines with suppressed [171] or ablated ApoE expression [82]. Also, core protein accumulated at the lipid droplet surface of the double ApoB/ApoE knockout Huh-7 cells [82], although no obvious difference in core localization was observed upon ApoE complementation of the 293T cell line [122]. Altogether, ApoE seems to participate in the virion maturation and to confer the infectivity. Despite the technical challenge, it would be worth examining the density of the presumed non-infectious intracellular particles produced in absence of ApoE. Infectious intracellular virions were previously shown to have a higher density as compared to the secreted virions, suggesting a post-assembly maturation step during virus release [18] and it would be interesting to test whether this density shift occurs in absence of ApoE.

The secretability of ApoE and its amphipathic α-helices are crucial for its proviral effect. Indeed, ApoE fusion to an ER retention signal prevented the protein secretion and concomitantly infectious HCV virions were retained in the cell [172]. Furthermore, ApoE N- or C-terminal deletion mutants retaining at least one of the α-helices are able to support virus production [82]. Finally, physical interaction was reported between ApoE and several HCV proteins, namely NS5A [9], NS2 [171] and the envelope glycoprotein E2 [171]. The pertinence of the first interaction is a matter of discussion due to the incompatible topologies of the two partners and is undermined by the plethora of described NS5A interactors [9]. Furthermore, ApoE binding to NS2 and NS5A is weak [171]. On the opposite, ApoE interaction with E2 resists to high stringency buffers (1% Triton X-100) and to the dissociation of cholesterol-rich lipid microdomains by saponin [171,173]. Importantly E2 transmembrane domain mediates both the recruitment of the E1E2 glycoprotein complex to cholesterol-enriched detergent-resistant lipid raft membranes and its interaction with ApoE [171].

The growing numbers of proteins that can substitute for ApoE in HCV production (here called «ApoE mimics») seem to challenge the role of apolipoproteins. Nonetheless, no infectious HCV is produced in absence of apolipoprotein—or apolipoprotein substitute. With the observed redundant role of the exchangeable apolipoproteins and of ApoB in HCV production (see above, Section 4.4), it is likely that all apolipoproteins share a similar mechanism of action. The panel of ApoE mimics further offers the unique possibility to better distinguish which of the above-mentioned ApoE functions, motifs and interactions are really relevant to HCV production. Thus, like ApoE, CAMP restores the correct core localization, which tended to accumulate at the lipid droplet surface in absence of apolipoproteins, and increases HCV specific infectivity [135]. E^rns^ and NS1 reproduce the ApoE participation in intracellular virion assembly [143]. The two proteins incorporate in the virion and increase its specific infectivity, suggesting a role in infectious particle maturation or entry [143]. Intriguingly, the particles harboring ApoE, CAMP, E^rns^ or NS1 were undistinguishable in terms of density, at least at the resolution of the assay [135,143]. Furthermore, at least NS1 and E^rns^ are likely to boost virus entry via interaction with cell surface heparan sulfates [148,153]. Finally, antibacterial peptides, including LL-37, also bind heparin and glycosaminoglycans, so that they may also complement ApoE functions during HCV entry [141].

A list of chimeras and mutants of ApoE or ApoE mimics have been tested for their ability to rescue HCV production [82,117,135,143]. In short, in all tested chimeras (based on ApoE, ApoC-I, CAMP and E^rns^), the proviral function can be pinpointed to amphipathic α-helices [82,117,135,143]. Consistently, single-point helix-breaking mutations abolished ApoC-I proviral effect [117]. In the case of ApoE, ApoC-I or E^rns^, one single α-helix can be enough to support HCV production [82,143]. If E^rns^ single α-helix is long and tilted (see above) [146] (Figure 5f), this is not the case of the ApoC-I individual helices [82,117]. CAMP, however, needs its two amphipathic helices (14 and 17 residues separated by a lysine residue) to mediate HCV assembly [135]. Of all ApoE mimics, NS1 is possibly the most unexpected on a structural point of view given its predominant beta-sheet structure [149]. Mutagenesis on this protein will be important to test whether not only unrelated protein sequences but also distinct protein structures can substitute for apolipoproteins in HCV production.

So what exact function do apolipoproteins, E^rns^, NS1, CAMP and possibly other proteins fulfill in HCV assembly that goes beyond the competence of the virus own proteins? First of all, all described apolipoprotein mimics are secreted proteins [82,117,135,143]. Like apolipoproteins, E^rns^ and NS1 follow the conventional secretion pathway and travel through the Golgi apparatus, as attested by their glycosylation profile [174,175]. A non-secreted version of ApoE is not able to mediate HCV production [172]. What is more, most apolipoprotein mimics are able to recruit a lipid cargo. This is the case of the apolipoproteins and of SAA, but also of NS1, which forms barrel-shaped HDL-like particles [151]. Whether E^rns^ or CAMP are really secreted as soluble proteins or as lipid-bound structures could be investigated in more details. These two properties, secretability and lipid loading, suggest that apolipoproteins or their substitutes could carry HCV as a cargo through the secretory pathway.

It is still not fully clear whether HCV envelope glycoproteins E1 and E2 can trigger the virion budding [83]. The budding force does seem to come from the envelope shell, since HCV E1E2 were shown, in absence of any other virus component, to associate with lipoproteins in empty virus-like particles [176]. For many viruses, lateral interactions between the envelope glycoproteins generate a pushing or pulling force that drives budding [177]. The resulting virions often exhibit a very structured and symmetrical protein lattice on their envelope. In the case of HCV, no such structure has been visualized by electron microscopy [15,16,17]. Yet, virion-embedded E1E2 glycoprotein heterodimers are organized in high-molecular weight protein complexes maintained by disulfides [74]. These intermolecular disulfide bonds could promote a local driving force initiating budding. Alternatively, apolipoproteins or mimics could provide the budding force. Indeed, amphipathic α-helices can induce membrane curvature already by inserting in the phospholipid layer [178]. Several of the helices that can rescue HCV production have further structural elements that could potentially bend membranes: e.g., the tilt of the E^rns^ helix [146], the hinge between consecutive helices in ApoC-I or CAMP [117,135], or multiple helices where electrostatic helix-to-helix lateral interactions might favor a lattice formation and membrane bending [179,180]. Note, however, that a role of apolipoproteins or mimics in HCV budding is in contradiction to the correct envelopment of HCV virion in absence of apolipoproteins [82,122,171]. It cannot be excluded that mechanical shearing of the cells in the envelopment assay increases the envelopment of partially enwrapped virions.

An interaction between the apolipoproteins and HCV envelope might help to coordinate the incorporation of both elements in the hydrid lipo-viro-particle. But if ApoE was shown to interact with E2 [171,173], there is no such report for the other apolipoproteins nor for NS1, E^rns^, or CAMP. Note however that the pestiviral E^rns^ interacts with its cognate E2 and is incorporated in the virion [181]. Given that the ApoE mimics do not share sequence homology, a broad interaction spectrum would however be surprising. Possibly, in the artificial overexpression systems used, the protein quantity of the ApoE mimics is such that an interaction with HCV E2 is not crucial to mediate protein encounter and virion assembly.

Disulfide bond reshuffling might also cause the incorporation of apolipoproteins in the large covalent E1E2 complexes. The mere size of ApoB (550 kDa) could account for the large size of these astonishing structures [74]. ApoB forms 8 disulfides that are important for the protein folding and secretion [182]. Furthermore, both ApoE, at least in its major ApoE3 allele, and ApoB contains several cysteines (up to 2 for ApoE and 25 for ApoB). ApoE disulfide bond formation accepts some flexibility as it maintains ApoE3 homodimers as well as ApoE3-ApoA-II heterodimers [183]. Of note however, the three major ApoE isoforms differ in cysteine abundance (2 for ApoE2, 1 for ApoE3 and none for ApoE4, in the mature protein) [184] and yet can support HCV production without distinction [121,185,186,187]. NS1 C-terminus has 6 cysteines which form disulfide bonds and are possibly involved in dimer formation [150]. E^rns^ has 9 conserved cysteines in its ectodomain, one of them establishes an interchain disulfide that stabilizes the E^rns^ homodimer [188].

Finally, if one can rather easily imagine the association of HCV virion within a mixed lipoprotein structure incorporating apolipoproteins, it remains unclear how HCV and NS1 HDL-like particles could intermingle. The NS1 symmetrical hexamer [149,151] seems so rigid that it is unlikely to accommodate virion components such as a capsid and even less HCV envelope glycoproteins (Figure 5g). The fact that NS1 can support HCV production rather argues for a two-particle model where immature non-infectious HCV intracellular virions latch onto lipoproteins or lipoprotein-like structures and are taken up as cargos in the secretory pathway. Alternatively, NS1 might be incorporated in another oligomeric state in the trans-complemented HCV particle. In this context, the artificial NS1-rescued HCV virion would be an interesting subject for electron microscopy analysis after HCV-specific immunocapture. More generally, a deeper characterization of the virion assembly and biophysical properties when apolipoproteins are replaced by these unrelated proteins (flaviviral NS1, pestiviral E^rns^ or CAMP) will be of great interest. First of all, it will be important to test whether these apolipoprotein mimics also engage interactions with HCV E2 glycoproteins, so as to understand their mode of incorporation in the virion and determine their possible role in budding. Secondly, and although no difference in virion density profile was observed so far [135,143], it will be intriguing to evaluate the lipidome of these artificial virions and to verify whether they do form genuine lipo-viro-particles. Most importantly, the impact of the apolipoprotein mimics on antibody-mediated neutralization remains to be investigated. It might be that the apolipoprotein mimics allow virus production without protecting the virion from neutralization, in which case they might be of help for the design of more antigenic HCVcc-based inactivated vaccines [189].

## 9. Conclusions

The reason HCV has been eluding microscopy observation is possibly also the reason why it so efficiently escapes the host antibody response and presents such a hurdle to vaccine design: HCV strongly resembles physiological VLDL particles. This mimicry implies convergences in the biogenesis of the virus and the host VLDL, which likely allows HCV to incorporate neutral lipids, to acquire its apolipoprotein coat, and to find its way out of the infected cell. The recent discovery that lipoprotein-unrelated proteins can substitute for apolipoproteins in HCV assembly, release and entry [135,143] is too recent to fully understand its imprint on our picture of HCV morphogenesis. It certainly opens new research avenues in term of possible extra-hepatic HCV reservoir and in the hunt for a vaccine. A major impediment in understanding HCV assembly is the lack of a full understanding of the VLDL secretion pathway. While lipoproteins quantitatively and functionally are a major component of our plasma and metabolism, so far, only models are available to explain their production. Strong imaging data, specific purification procedures and cellular systems reproducing as closely as possible the hepatic characteristics are missing to fulfill the picture. Joint efforts between cell biologists, microscopists, and virologists will be instrumental to deepen our understanding of the host machinery supporting HCV production. Unravelling this unique interaction between HCV and its host lipid metabolism might also reveal new genetic determinants of the host susceptibility to HCV infection. Finally, the nature of HCV lipo-viro-particle represents a challenge for vaccine design and will certainly motivate new vaccine concepts and platforms.
